# Comparison of Using Second-Generation Cryoballoon and Radiofrequency Catheter for Atrial Fibrillation Ablation in Patients With the Common Ostium of Inferior Pulmonary Veins

**DOI:** 10.3389/fcvm.2021.794834

**Published:** 2022-01-11

**Authors:** Jia-hui Li, Hai-yang Xie, Qi Sun, Xiao-gang Guo, Yan-qiao Chen, Zhong-jing Cao, Jian Ma

**Affiliations:** State Key Laboratory of Cardiovascular Disease, Arrhythmia Center, National Center for Cardiovascular Diseases, Fuwai Hospital, Chinese Academy of Medical Sciences and Peking Union Medical College, Beijing, China

**Keywords:** catheter ablation, cryoballoon, atrial fibrillation, pulmonary vein, common ostium

## Abstract

**Aims:** To compare the procedural outcomes of cryoballoon ablation (CBA) and radiofrequency ablation (RFA) in atrial fibrillation (AF) patients with the common ostium of inferior pulmonary veins (COIPV) and to explore the effect of COIPV on CBA performance through the assessment of anatomical factors.

**Methods:** A total of 18 AF patients with COIPV were included. Pulmonary vein isolation (PVI) was performed with second-generation CBA or RFA. The anatomical characteristics of COIPV and procedural outcomes were collected.

**Results:** The prevalence of COIPV was 0.82% in the enrolled population. PVI was achieved in all pulmonary veins (PVs) without any complications. The “tricircle” strategy was applied for RFA, and the segmental freeze strategy was performed for CBA. Compared with RFA, CBA had shorter procedural time (median: 53.0 vs. 78.0 min, *p* < 0.001) and longer fluoroscopy time (median: 13.5 vs. 6.0 min, *p* < 0.001). Higher ovality index of the ostium was seen in patients with ≥4 freezes in inferior PVs [IPVs; 0.95 (0.78–1.05) vs. 0.49 (0.21–0.83), *p* = 0.047]. During a median of 23.5 months of follow-up, the atrial arrhythmias-free survival after the procedure was comparable between CBA and RFA (*p* = 0.729).

**Conclusion:** The second-generation CBA is an efficient and safe alternative for RFA in AF patients with COIPV. Anatomical characteristics of COIPV bring the challenge to the procedure performance of RFA and CBA.

## Introduction

Atrial fibrillation (AF) is a prevalent cardiac arrhythmia, which affects ~1–2% of the entire population (1). Pulmonary vein isolation (PVI) has been recognized as the cornerstone of catheter ablation for AF treatment (2, 3). The common ostium of inferior pulmonary veins (COIPV) is an unusual variation of pulmonary venous drainage, whose anatomical characteristics and electrophysiology are rarely demonstrated. For AF ablation in patients with COIPV, the feasibility and safety of cryoballoon ablation (CBA) have been firstly reported by Xie et al. (4). However, studies comparing radiofrequency ablation (RFA) and CBA have been scarcely reported, and no data on the relationship between anatomical factors of COIPV and procedural difficulty of CBA are available.

This study compares the procedural outcomes of PVI using CBA and RFA in AF patients with COIPV and to explore the effect of COIPV on CBA performance through the anatomical data of COIPV.

## Methods

### Study Population

Building on the study of Xie et al. (4), the current study expanded the time span and the sample size of enrollment. Therefore, in a total population of 2,201, patients with AF referred for index PVI between October 2013 and October 2019, 18 consecutive patients (0.82%) with COIPV were retrospectively analyzed. Ten patients underwent CBA as Xie et al. reported, and the remaining 8 underwent RFA. The presence of COIPV was determined by the multidetector CT (MDCT) scanning and a three-dimensional reconstruction cardiac image (Supplementary Figure 1). All the enrolled patients had clinically documented paroxysmal or persistent AF. All the procedures were performed by an experienced electrophysiologist who had the experience of 300 AF ablations (including more than 120 CBAs) per year, and the ablation approach was selected based on preference of the operator.

This study was conducted in accordance with the Declaration of Helsinki (as revised in 2013) and was approved by the Ethics Committee of Fuwai Hospital. The written consent was obtained from each patient.

### Mapping and Ablation Protocol

Before the procedure, antiarrhythmic drugs (AADs) were discontinued for at least five half-lives, and the transesophageal echocardiography was routinely performed to exclude the presence of intracardiac thrombi. All procedures were performed under moderate sedation with continuous infusion of midazolam and fentanyl. Heparin was administered based on 100 IU/kg body weight, and the activated clotting time was maintained at 300–350 s. The decapolar catheter was positioned in the coronary sinus through the right internal jugular vein. For patients who underwent CBA, the quadripolar catheter was placed at the right ventricular apex via femoral venous access. Electrophysiological data were continuously recorded by a multichannel system (LabSystem PRO, Bard Electrophysiology, Lowell, MA, USA). The bipolar electrograms were filtered between 30 and 500 Hz.

At the end of the procedure, sinus rhythm was restored by 200J cardioversion if AF continued. Then, all patients underwent a 30-min observing period in sinus rhythm for the evaluation of PVI in all PVs. If there was no evidence of reconnection between the left atrium (LA) and pulmonary vein (PV) during the observing period, then acute success was considered, otherwise an extra CBA or RFA was repeated for the reconnected PV.

### Radiofrequency Ablation

A three-dimensional electroanatomic mapping system (CARTO, Biosense Webster Inc., Diamond Bar, CA, USA) was utilized as an RFA approach. A decapolar Lasso catheter (Biosense Webster Inc., Diamond Bar, CA, USA) was placed at the PV ostium to identify PV potentials (PVPs). The circumferential PVI was performed with irrigated contact force ablation catheter (THERMOCOOL SMARTTOUCH, Biosense Webster Inc., Diamond Bar, CA, USA), and the ablation was guided by local near-field electrograms rather than ablation index. The radiofrequency (RF) energy was applied 25 W except for 30–35 W at the anterior aspect of LA and 20 W at the posterior wall. During RF delivery, the contact force was kept between 15 and 20 g, with a saline irrigation rate of 17 ml/min and a temperature limit of 43°C. The “tricircle” ablation (5) was performed with three ablation circles surrounding the left superior pulmonary vein (LSPV) ostium, right superior pulmonary vein (RSPV) ostium, and the common ostium of inferior PVs (IPVs). The endpoint of ablation was electrical PVI confirmed by Lasso catheter in PVs.

### Cryoballoon Ablation

The CBA was performed using a 28-mm CB catheter (Arctic Front Advance, Medtronic, Inc., Minneapolis, MN, USA) inserted through a 12 F steerable sheath (FlexCath, Medtronic, Inc., Minneapolis, MN, USA). Real-time PVPs and electrical PVI were recorded using a 20-mm diameter inner lumen mapping catheter (Achieve; Medtronic, Inc., Minneapolis, MN, USA). Optimal PV occlusion was achieved with the “proximal-seal” technique (6). If an optimally full occlusion was hard to achieve, the segmental freezing strategy would be adopted. As previously described (7), time to isolation (TTI) was determined by the complete disappearance of PVPs or dissociation between PVPs and LA electrical activity. Each cryoablation was delivered 180 s, and a bonus cryoablation was needed for the PV if TTI was more than 60 s. If real-time PVP monitoring and TTI were not recorded clearly, the Achieve catheter would be advanced into PV ostium to confirm PVI after freezing. If the nadir temperature during freezing was lower than −60°C, the cryoablation would be terminated immediately. In the case of phrenic nerve palsy during cryoablation in right PVs, high-output diaphragmatic stimulation (1,500 ms, 20 mA) was performed with a quadripolar catheter within the superior vena cava.

The total number of freezes in left IPV (LIPV) and right IPV (RIPV) was acquired to divide the patients with CBA into two subgroups: subgroup A with 1–3 freezes and subgroup B with ≥4 freezes.

### Anatomy Definition

The PV ostium was defined as the visual intersection of PV and LA. Based on the three-dimensional reconstruction image, the anatomy definition of COIPV was that the two IPVs converged and shared one common ostium near the midline of the LA posterior wall (8). As previously reported (5), the COIPV could be further identified as type A without a short common trunk and type B with a short common trunk. In MDCT scanning, the distance from the esophagus to IPV <5 mm suggested a close contact between the two structures (9).

### Diameter and Angle Measurements

**Figure 1:**
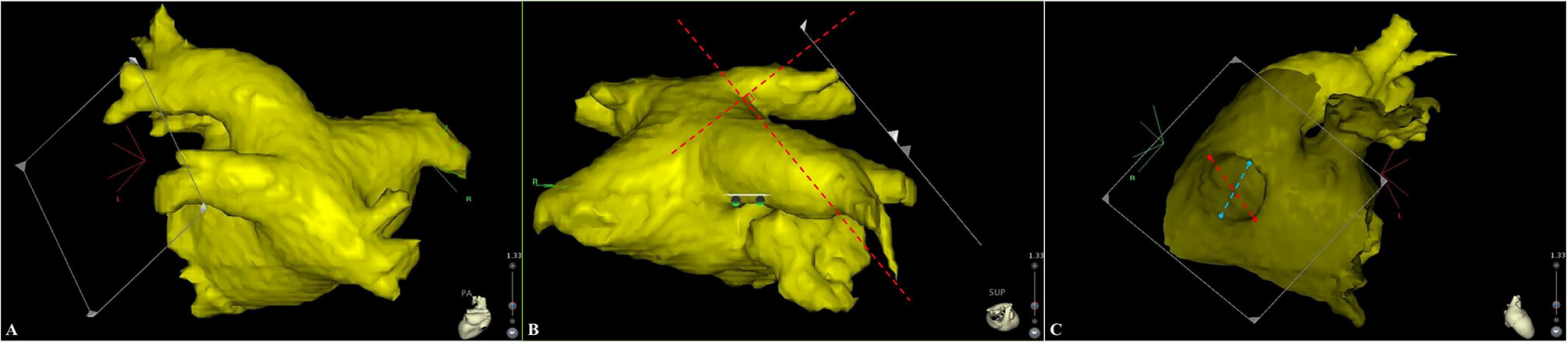
A strictly perpendicular plane to the pulmonary ostium **(A,B)** was used for measurement of the maximal and minimal diameters **(C)**. The red and blue dotted lines represent for the maximal and minimal diameter, respectively.

**Figure 2:**
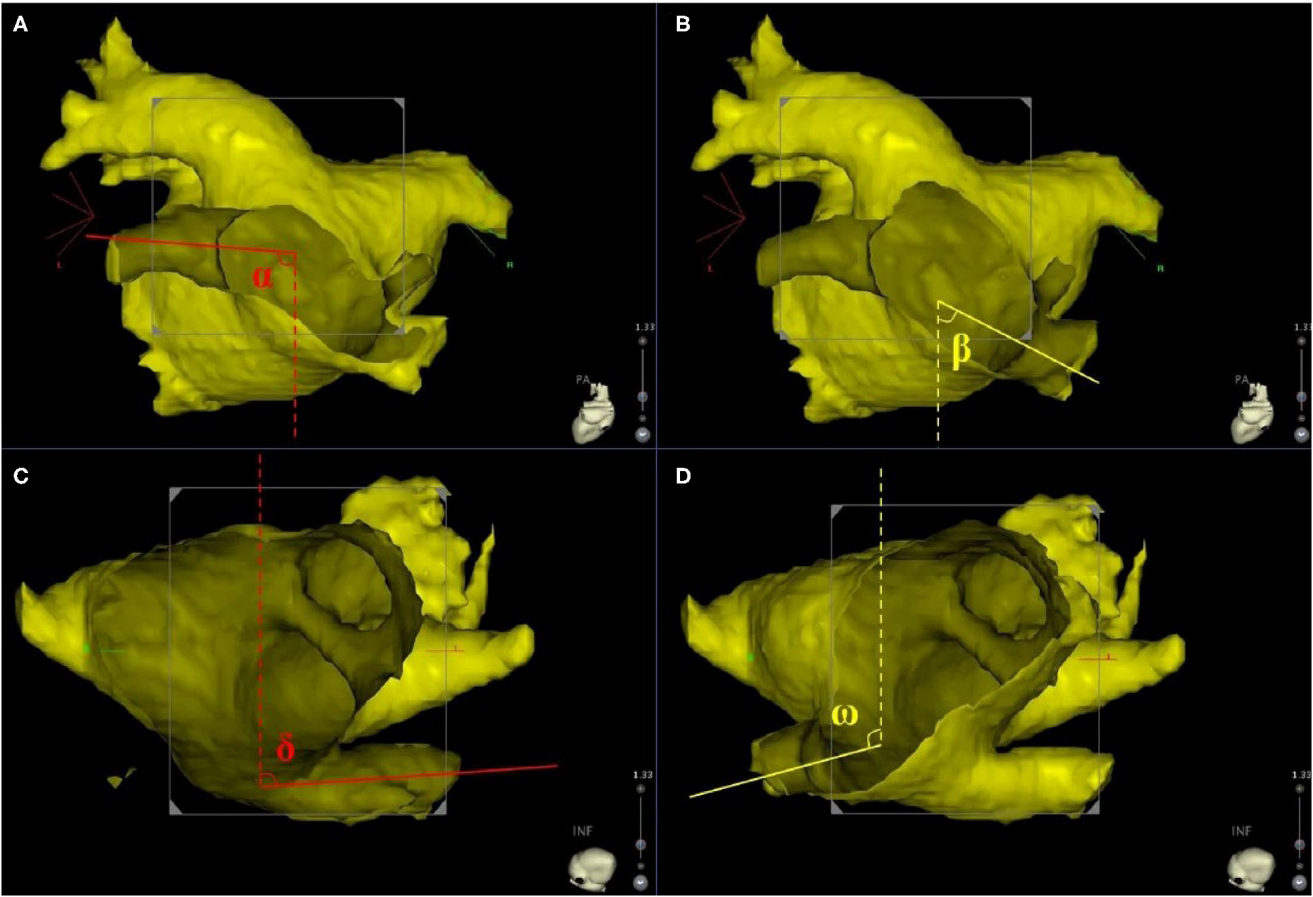
The principle axis of LIPV (red line) and RIPV (yellow line) were identified in frontal and transverse planes. **(A,B)** Display the frontal orientation measurements of LIPV and RIPV, resectively. Angles α and β represent for the angles between LIPV, RIPV, and the sagittal plane reference (dotted line) in the frontal plane. **(C,D)** display the transverse orientation measurements of LIPV and RIPV, resectively. Angles δ and ω represent for the angles between LIPV, RIPV, and the sagittal plane reference (dotted line) in the transverse plane. LIPV, left inferior pulmonary vein; RIPV, right inferior pulmonary vein.

The three-dimensional reconstruction cardiac image of each patient was acquired using CartoMerge software (Biosense Webster, Diamond Bar, CA, USA). The images were analyzed by two observers who were blinded to the study. Diameter measurements were performed with digital calipers in CartoMerge software. As previously described (10), a strictly perpendicular plane to the pulmonary ostium was used for measurement of the maximal and minimal diameters (Figure 1). The ovality index of PV ostium was 2 × (maximal diameter–minimal diameter)/(maximal diameter + minimal diameter). Angle measurements were performed using a digital protractor in the screen snapshot, and the anatomical characteristics of LIPV and RIPV were assessed as follows: two lines were drawn as the principal axis of LIPV and RIPV, respectively. Then, the angles between the principal axis of LIPV/RIPV and a reference line of the sagittal plane were measured in both transverse and frontal planes (Figure 2).

### Post-procedural Management

After returning to the ward, all patients routinely underwent transthoracic echocardiography to exclude pericardial effusion and tamponade. Continuous ECG monitoring was performed for at least 24 h until discharge from the hospital. Pantoprazole was prescribed for at least 2 months. The oral anticoagulants were continued for at least 2 months, thereafter, an anticoagulant strategy corresponding to individual CHA_2_DS_2_-VASc score [congestive heart failure (1 point), hypertension (1 point), age ≥75 years (2 points), diabetes mellitus (1 point), prior stroke or transient ischemic attack (2 points), vascular disease (1 point), age 65 to 74 years (1 point) and female (1 point)] was adopted. Patients with persistent AF received AADs continuously for 3 months to maintain sinus rhythm and visited the outpatient for assessment of drug reactions. Discontinuation of AADs was recommended if the patient was free from AF.

### Repeat Ablation Procedure

All patients with evidence of atrial arrhythmias (AAs) recurrence were suggested repeat procedure with the three-dimensional electroanatomic mapping system (CARTO, Biosense Webster Inc., Diamond Bar, CA, USA). The recurrence of PV-LA conduction was confirmed by the decapolar Lasso catheter (Biosense Webster Inc., Diamond Bar, CA, USA). RFA for PV gaps was performed with irrigated contact force ablation catheter (THERMOCOOL SMARTTOUCH, Biosense Webster Inc., Diamond Bar, CA, USA). PVI was the endpoint of the redo procedure. If a macro-reentry could not be excluded, three-dimensional activation electroanatomic mapping and entrainment technique were performed to determine the reentry circle and isthmus. The bidirectional block was achieved after linear ablation of the isthmus.

### Follow-Up

All patients were followed up at 1, 3, 6, 9, and 12 months after the procedure and every 6 months thereafter. Each time 12-lead ECG or 24-h Holter recordings were suggested to monitor rhythm status. Outpatient records and subsequent diagnoses were collected using an electronic medical recording system and telephone interviews. Recurrence was defined as any evidence of AAs with a duration longer than 30 s beyond a 3-month blanking period after ablation.

### Statistical Analysis

Continuous data were expressed as the median (25th, 75th percentiles) and were compared using Mann-Whitney *U*-test. Categorical variables were expressed as percentages and were compared using the Chi-square test or Fisher's exact test when appropriate. The k statistic was used to evaluate the interobserver agreement (IOA) in the anatomical parameters of COIPV. Kaplan–Meier method and log-rank tests were performed to compare AAs-free survival. All statistical tests were two-sided, and statistical significance was defined as *p* < 0.05. All analyses were performed using SPSS 24.0 (SPSS Inc., Chicago, IL, USA).

## Results

### Baseline Characteristics

Among the 18 enrolled patients, the median age was 57.0 years, and the majority were male (88.9%). There was no significant difference in baseline characteristics between RFA and CBA groups (Table 1). The MDCT scanning showed that in 8 patients, the esophagus was located close to the LIPV. In 3 of the 8 patients, the esophagus was exposed to the posterior junction part of the two IPVs, and in the remaining 5 patients, the esophagus stayed near the main branch of LIPV but away from the ostium. In all 18 cases, no aorta was close to the IPV (Supplementary Figure 2).

**Table 1:** Baseline characteristics of the study population.

	**Total**	**CBA**	**RFA**	***p*-value**
*N*	18	10	8	
Paroxysmal AF, *n* (%)	15 (83.3)	9 (90.0)	6 (75.0)	0.832
Previous AF ablation, *n* (%)	0	0	0	–
Age, years	57.0 (49.8, 63.3)	56.5 (47.0, 61.5)	59.5 (53.5, 66.3)	0.274
Male, *n* (%)	16 (88.9)	9 (90.0)	7 (87.5)	1.000
BMI, kg/m^2^	26.2 (24.3, 28.2)	26.4 (24.9, 27.4)	24.7 (23.5, 28.5)	0.573
Hypertension, *n* (%)	4 (22.2)	2 (20.0)	2 (25.0)	1.000
Diabetes mellitus, *n* (%)	0	0	0	–
Heart failure, *n* (%)	0	0	0	–
CHA_2_DS_2_-VASc score	0 (0, 1.0)	0 (0, 1.0)	0.5 (0, 1.0)	0.762
Antiarrhythmic medication	3 (16.7)	1 (10.0)	2 (25.0)	0.832
Left atrial diameter, mm	40.5 (38.0, 42.3)	41.5 (40.0, 43.3)	39.0 (33.5, 41.5)	0.068
LV ejection fraction, %	63.0 (60.0, 65.0)	63.5 (61.5, 65.5)	61.0 (60.0, 64.8)	0.408
Recurrence, *n* (%)	3 (16.7)	2 (20.0)	1 (12.5)	1.000
Follow-up time, months	23.5 (10.5, 31.8)	24.0 (13.8, 31.0)	22.0 (9.5, 34.8)	1.000

*Data are presented as median (25th, 75th percentiles) or n (%)*.

*AF, atrial fibrillation; BMI, body mass index; CBA, cryoballoon ablation; LV, left ventricle; PAF, paroxysmal atrial fibrillation; RFA, radiofrequency ablation*.

The PVI was successfully achieved in all PVs without complication. Compared with RFA, CBA had shorter procedural time (median: 53.0 vs. 78.0 min, *p* < 0.001) and longer fluoroscopy time (median: 13.5 vs. 6.0 min, *p* < 0.001; Table 2).

**Table 2:** Procedural and fluoroscopy time of the study population.

	**CBA**	**RFA**	***p*-value**
Procedure time, minutes	53.0 (42.0, 61.0)	78.0 (75.0, 85.3)	<0.001
Fluoroscopy time, minutes	13.5 (11.8, 16.5)	6.0 (5.0, 7.0)	<0.001

*Data are presented as median (25th, 75th percentiles)*.

*CBA, cryoballoon ablation; RFA, radiofrequency ablation*.

### Procedural Characteristics of Cryoablation

**Figure 3:**
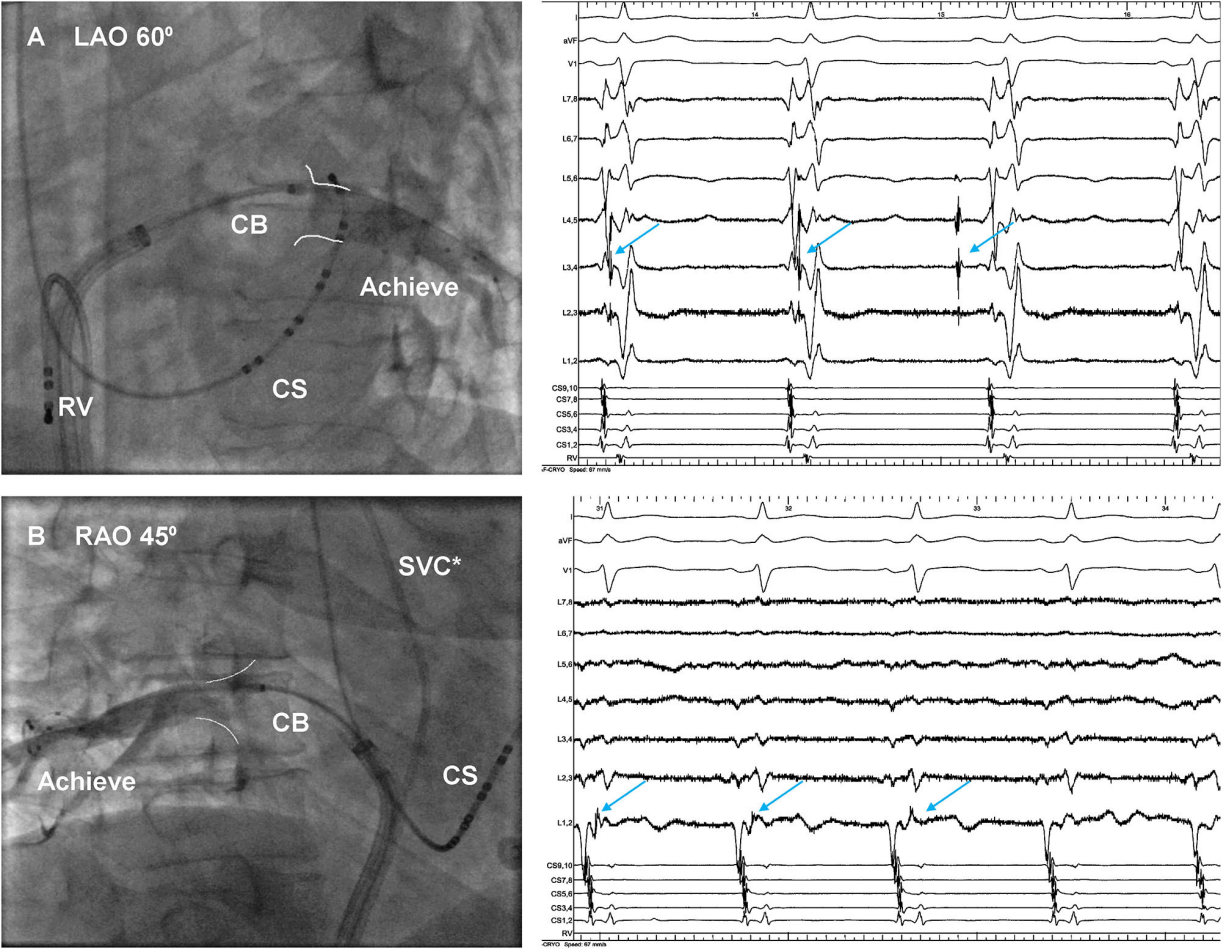
Venography of inferior pulmonary veins during cryoballoon (CB) ablation. The 28-mm CB was positioned at **(A)** ostium of the left inferior pulmonary vein and **(B)** ostium of the right inferior pulmonary vein. The conduction of PVPs (arrow) was delayed and then blocked. *The quadripolar catheter was placed within SVC. CS, coronary sinus; RV, right ventricle; SVC, superior vena cava; PVPs, pulmonary vein potentials.

A total of 10 patients with 40 PVs underwent CBA with second-generation CB, and the procedural parameters were displayed in Table 3. Optimally full occlusion for common ostium was hard to achieve by the 28 mm CB, thus, the segmental freezing strategy was adopted in all 10 patients (Figure 3), and PVI was achieved in all IPVs without touching up. A median number of 3, 2, 2, 2 freezes was applied in LSPV, LIPV, RSPV, and RIPV, respectively, and each patient had a median total number of 4 freezes in both IPVs. There was no significant difference in procedural characteristics between subgroups A and B except the total number of freezes in IPVs.

**Table 3:** Procedural characteristics of CBA group.

	**Total**	**Subgroup A**	**Subgroup B**	***p*-value**
*N*	10	4	6	
Total number of freezes in IPVs	4.0 (3.0, 4.3)	3.0 (3.0, 3.0)	4.0 (4.0, 5.3)	0.010
**LSPV**				
Number of freezes, *n*	3.0 (2.0, 3.3)	2.5 (2.0, 3.0)	3.0 (2.0, 4.3)	0.476
Time to isolation, seconds	41.5 (35.3, 45.3)	41.5 (37.8, 43.0)	40.5 (30.0, 54.8)	1.000
Temperature at isolation, °C	−34.5 (−32.0, −39.5)	−33.0 (−32.0, −39.3)	−37.0 (−32.3, −39.5)	0.610
Nadir temperature, °C	−47.5 (−42.0, −54.5)	−46.5 (−42.3, −55.3)	−47.5 (−40.5, −54.5)	0.762
**LIPV**				
Number of freezes, *n*	2.0 (1.0, 2.0)	1.0 (1.0, 1.8)	2.0 (2.0, 2.0)	0.067
Time to isolation, seconds	51.5 (26.8, 78.3)	40.0 (26.3, 72.5)	55.0 (32.0, 80.8)	0.762
Temperature at isolation, °C	−33.0 (−27.3, −38.5)	−29.0 (−25.8, −35.3)	−36.5 (−26.5, −40.3)	0.257
Nadir temperature, °C	−39.5 (−35.0, −46.5)	−39.5 (−35.8, −46.3)	−39.0 (−35.0, −47.3)	1.000
**RSPV**				
Number of freezes, *n*	2.0 (1.8, 3.3)	2.5 (1.3, 6.8)	2.0 (1.8, 3.3)	0.762
Time to isolation, seconds	47.0 (40.8, 63.5)	47.0 (31.0, 51.8)	47.0 (41.5, 108.5)	0.610
Temperature at isolation, °C	−40.5 (−35.5, −46.3)	−36.5 (−30.0, −39.3)	−45.0 (−39.3, −47.5)	0.067
Nadir temperature, °C	−53.0 (−49.8, −54.5)	−53.0 (−48.3, −55.5)	−53.0 (−49.8, −54.5)	1.000
**RIPV**				
Number of freezes, *n*	2.0 (2.0, 2.3)	1.8 (1.3, 2.0)	2.0 (2.0, 3.3)	0.257
Time to isolation, seconds	48.5 (32.5, 55.0)	48.5 (43.8, 87.0)	45.0 (15.3, 55.0)	0.610
Temperature at isolation, °C	−36.0 (−26.8, −40.3)	−36.0 (−30.5, −43.0)	−34.5 (−18.3, −40.3)	0.610
Nadir temperature, °C	−45.5 (−40.8, −55.0)	−50.0 (−41.8, −54.5)	−43.0 (−39.8, −55.3)	0.762

*Data are presented as median (25th, 75th percentiles) or n*.

*CBA, cryoballoon ablation; IPVs, inferior pulmonary veins; LSPV, left superior pulmonary vein; LIPV, left inferior pulmonary vein; RSPV, right superior pulmonary vein; RIPV, right inferior pulmonary vein*.

### Anatomical Parameters of COIPV

The overall prevalence of COIPV was 0.82% in the population who underwent PVI in this study, and the short common trunk was a frequent presentation in 72.2% (13/18) patients with COIPV. The IOA in each measurement of the anatomical parameter was moderate or good (all *k* ≥ 0.8; Table 4). The anatomical parameters of COIPV in detail are shown in Table 5. No significant difference was seen between RFA and CBA. In further comparison between the two subgroups of CBA patients, a higher ovality index was seen in subgroup B who had a total number of ≥4 freezes in IPVs [0.95 (0.78–1.05) vs. 0.49 (0.21–0.83), *p* = 0.047].

**Table 4:** Interobserver agreement in anatomical parameters.

	**Total**	***k*-value**
**LIPV**		
Maximal diameter, mm	19.8 (17.8, 20.6)	0.805
Minimal diameter, mm	8.0 (6.5, 10.4)	0.812
Ovality index	0.83 (0.67, 0.94)	0.901
Frontal angle (α)	90.8 (88.6, 97.1)	0.939
Transversal angle (δ)	80.4 (75.8, 89.5)	0.846
**RIPV**		
Maximal diameter, mm	20.4 (18.2, 25.0)	0.890
Minimal diameter, mm	15.5 (12.0, 16.1)	0.779
Ovality index	0.42 (0.21, 0.56)	0.818
Frontal angle (β)	64.0 (49.2, 71.7)	0.929
Transversal angle (ω)	101.3 (95.2, 110.3)	0.871

*Data are presented as median (25th, 75th percentiles)*.

*LIPV, left inferior pulmonary vein; RIPV, right inferior pulmonary vein*.

**Table 5:** Anatomical parameters of COIPV.

	**RFA**	**CBA**	***p*-value**	**Subgroup A**	**Subgroup B**	***p*-value**
*N*	8	10		4	6	
Short common trunk, *n* (%)	7 (87.5)	6 (60.0)	0.444	2 (50.0)	4 (66.7)	1.000
**LIPV**						
Maximal diameter, mm	20.0 (19.0, 20.4)	18.5 (16.0, 21.6)	0.274	17.5 (11.5, 19.5)	19.5 (16.7, 24.2)	0.352
Minimal diameter, mm	8.5 (8.0, 9.8)	7.0 (6.2, 11.5)	0.573	9.2 (6.5, 13.4)	6.8 (5.4, 10.6)	0.476
Ovality index	0.81 (0.68, 0.89)	0.85 (0.51, 1.01)	0.965	0.49 (0.21, 0.83)	0.95 (0.78, 1.05)	0.047
Frontal angle	93.1 (89.4, 96.4)	90.0 (84.0, 98.9)	0.460	84.7 (79.5, 89.8)	95.6 (88.9, 99.2)	0.058
Transversal angle	78.0 (75.2, 80.5)	85.9 (76.7, 90.7)	0.203	85.9 (83.9, 94.1)	83.3 (72.7, 90.7)	0.610
**RIPV**						
Maximal diameter, mm	19.8 (18.5, 20.8)	23.2 (17.5, 26.6)	0.360	21.0 (18.6, 24.1)	25.8 (15.8, 27.5)	0.476
Minimal diameter, mm	16.0 (10.5, 16.0)	14.5 (12.5, 16.3)	0.965	12.8 (10.0, 15.8)	15.5 (13.5, 16.8)	0.257
Ovality index	0.31 (0.20, 0.58)	0.42 (0.34, 0.52)	0.696	0.42 (0.42, 0.67)	0.45 (0.16, 0.52)	0.610
Frontal angle	62.6 (47.5, 73.2)	64.0 (51.6, 71.7)	0.897	58.0 (47.2, 68.1)	68.4 (57.2, 76.3)	0.352
Transversal angle	106.7 (96.7, 110.8)	100.1 (95.1, 105.5)	0.408	97.7 (94.6, 119.7)	100.3 (96.0, 105.5)	0.762

*Data are presented as median (25th, 75th percentiles) or n (%)*.

*COIPV, common ostium of inferior pulmonary veins; CBA, cryoballoon ablation; RFA, radiofrequency ablation; LIPV, left inferior pulmonary vein; RIPV, right inferior pulmonary vein*.

### Follow-Up Results

**Figure 4:**
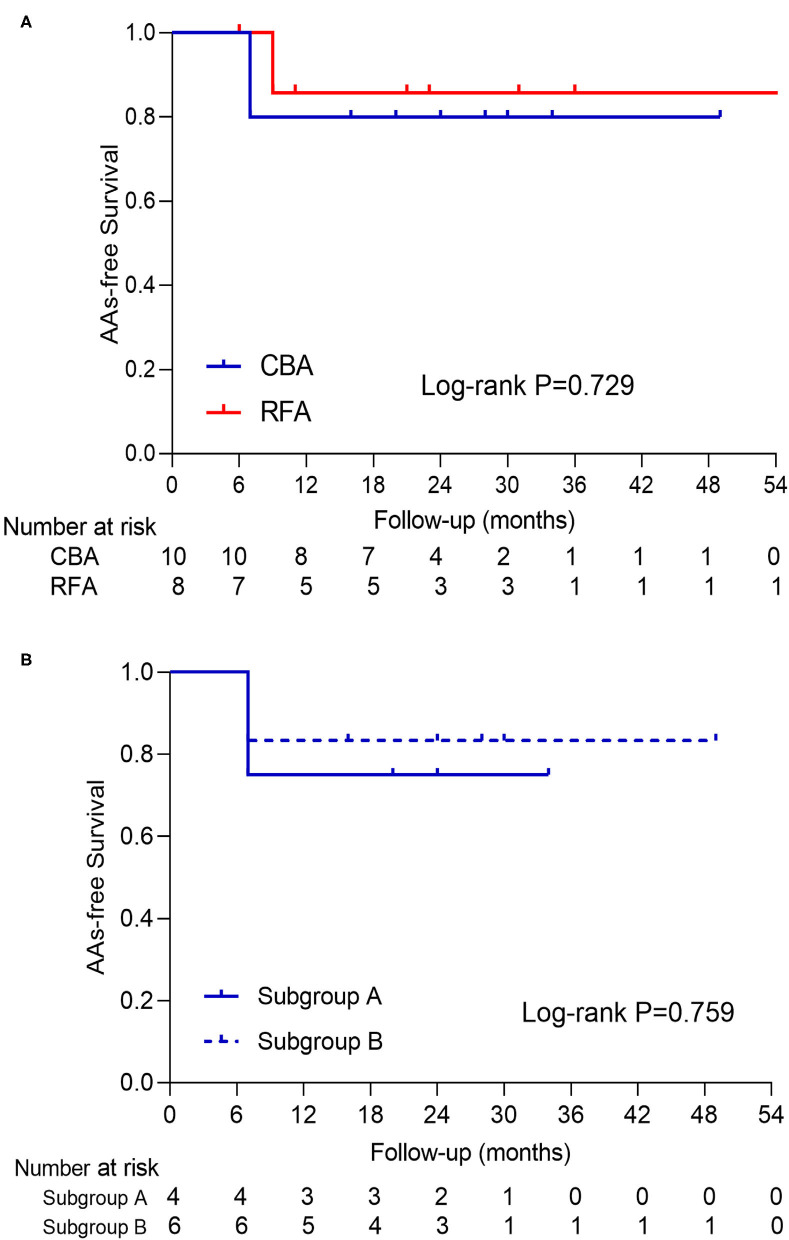
The presentation of AAs-free survival curve of patients in **(A)** CBA and RFA and **(B)** subgroup A and B. No log-rank *P*-value with statistical significance was seen in either curve. AAs, atrial arrhythmias; CBA, cryoballoon ablation; RFA, radiofrequency ablation.

No case was lost to follow-up in this study. All patients underwent rhythm monitoring regularly at 1, 3, 6, 9, and 12 months after the procedure and every 6 months thereafter. During a median of 23.5 months (interquartile range: 10.5–31.8 months) of follow-up, the AA-free survival after the procedure was comparable between CBA and RFA (Figure 4). The clinical outcome was also comparable at 12 and 18 months of follow-up (*p* = 0.083 and *p* = 0.541, respectively). Among CBA patients, the AA-free survival was similar between subgroups A and B (Figure 4).

**Figure 5:**
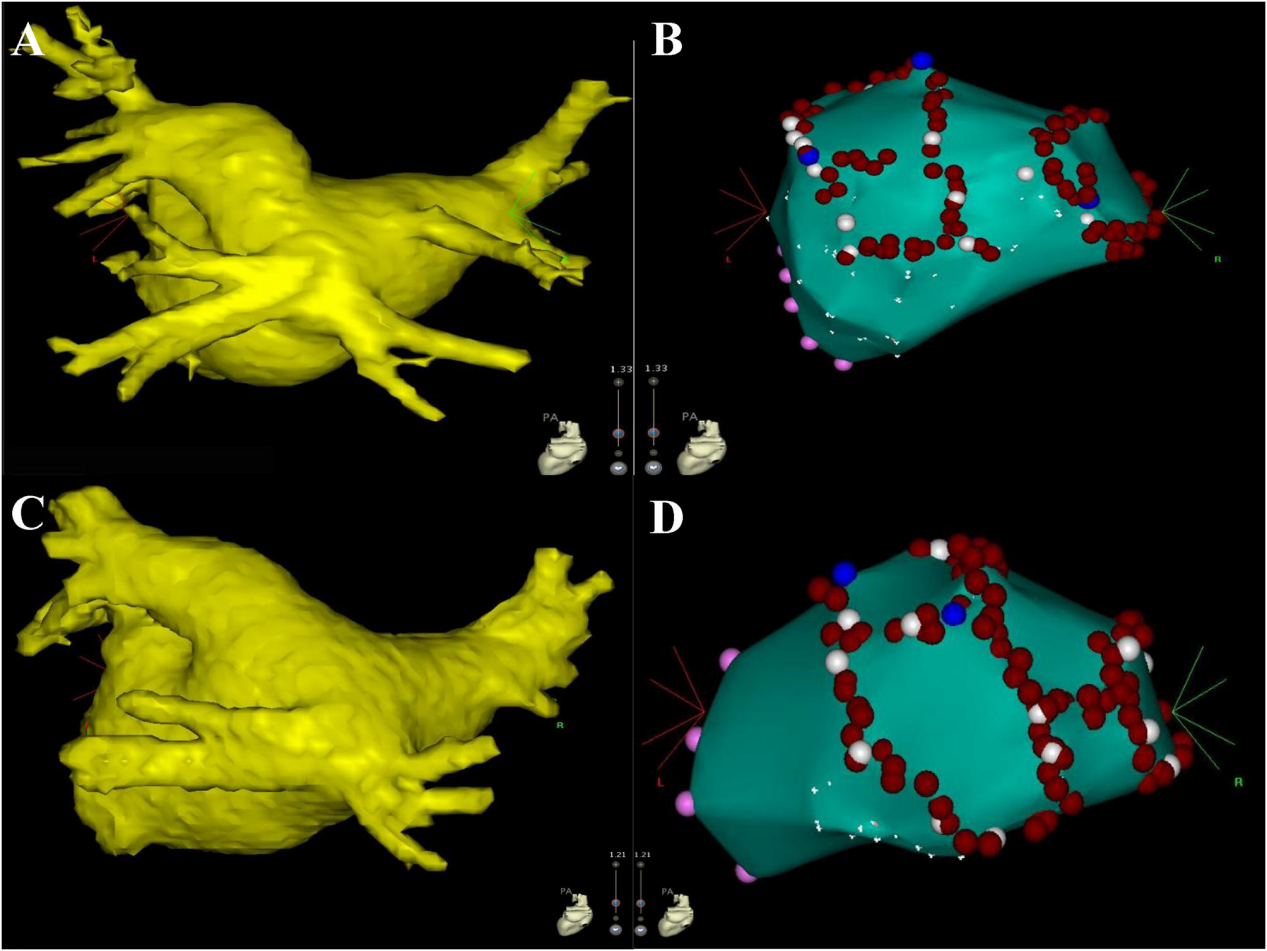
The figures display the results of the redo procedure. The left atrium, mitral valve (pink dots), and pulmonary venous ostium (white dots) were firstly identified with point-by-point mapping, then the conduction gaps were ablated successfully (blue dots). Part of ostium was also ablated (red dots) for consolidating PVI. Three conduction gaps were identified for patient #1 in the CBA group **(A,B)** the anterior segment, the roof of LSPV, and the posterior segment of RSPV. Two conduction gaps were identified for patient #14 in the RFA group **(C,D)** the anterior segment of LSPV and the contiguous site between LSPV and the common ostium. CBA, cryoballoon ablation; LSPV, left superior pulmonary vein; RSPV, right superior pulmonary vein; PVI, pulmonary vein isolation; RFA, radiofrequency ablation.

Of the 18 COIPV patients, 3 (16.7%) with paroxysmal AF before procedure had documented recurrence of AAs in 12-lead ECG and 24 h Holter: two in the CBA group had paroxysmal AF, and one in the RFA group had a combination of paroxysmal AF and typical atrial flutter (AFL). Except for one CBA patient who received conservative treatment, the rest two underwent RFA with point-by-point anatomical mapping in the redo-procedure, and 5 conduction gaps were identified: two sites in the anterior segment of LSPV, one in the roof of LSPV, one in the posterior segment of RSPV, and one contiguous site between LSPV and the common ostium (Figure 5). For the patient with typical AFL recurrence, RFA for cavotricuspid isthmus to bidirectional block was performed. No AA recurrence was presented in both patients after the redo procedure.

## Discussion

For AF ablation in patients with COIPV, the feasibility and safety of CBA have been reported in the previous study (4). The present study further compared the procedural outcomes between second-generation CBA and RFA and demonstrated the effect of COIPV on CBA performance by assessment of anatomical factors. The main findings are as follows: (1) the presents with COIPV in 0.82% of AF population who underwent PVI in this study; (2) comparing with RFA, CBA is a feasible technique with a similar procedural outcome and shorter total procedure time, despite lengthening total fluoroscopy time; and (3) the ovality of PV ostium and frontal angle of IPVs adds challenge to procedural performance in IPVs using 28 mm CB.

### Prevalence of COIPV

It is known that the right middle PV and common trunk of left PV are the two most common variations of PV drainage. The COIPV is a rare variation confirmed with MDCT and three-dimensional reconstruction image. Previously, Yu (5) reported the COIPV in 11 of 1,226 patients (0.9%) enrolled within 3 years. In Xie's et al. (4), the proportion of COIPV was 0.57%, which underestimated the actual prevalence because only CBA cases of COIPV were included in the final analysis. Building on Xie's study, the present study described 18 patients with COIPV who underwent CBA or RFA and reported COIPV in 0.82% of the enrolled population. Besides, the short common trunk was seen in a substantial proportion of COIPV (72.2%). Compared with other variations of PV drainage, the COIPV was so rare that data on prevalence was still scarce.

### Procedural Outcomes in Patients With COIPV

RF catheter has an advantage in point-by-point ablation with flexibility in fitting various anatomical structures of PV. However, for unusual variations, such as COIPV, the stability of the catheter tip becomes a big concern and a specific strategy for AF ablation should be considered. In Yu's et al. (5), eleven AF patients with COIPV underwent RFA with a “tricircle” ablation strategy. The PVI was achieved at a success rate of 90% without any complications. In the case reported by Squara (11), one AF patient with COIPV underwent RFA with one single ablation ring surrounding the entire LA posterior wall, to minimize the lesion on the posterior wall and to avoid esophageal injury. In the current study, Yu's “tricircle” ablation was successfully performed in all 8 patients in the RFA group without any complication, and AA-free survival during follow-up (7/8, 87.5%) was comparable with the result in Yu's study (10/11, 90.9%).

Previously, Xie et al. confirmed the feasibility and safety of second-generation CBA in performing PVI for patients with COIPV (4). The present study further confirmed that compared with RFA, using second-generation CBA in the setting of COIPV had similar long-term outcomes and shorter procedure time. Inevitably, CBA had a longer fluoroscopy time which resulted from confirming the CB occlusion in PVs by means of extra contrast injection and fluoroscopy. With the use of intracardiac echocardiography (ICE), the devices and cardiac structures, such as the FlexCath sheath, Achieve catheter, and the PV ostium, are easily visualized and navigated in the procedure, which reduces the need for fluoroscopy (12, 13). In recent years, a novel imaging and navigation system, the KODEX-EPD (EPD Solutions, Philips, Best, The Netherlands), has been used in CBA procedure. It gives a good three-dimensional image of cardiac structure and also allows to assess the real-time PV occlusion, reducing the use of fluoroscopy and contrast injection (14). In the future, the advantages of CBA in AF treatment will become more prominent with help of these new devices and systems.

### Procedural Characteristics in the Setting of COIPV

The uncommon morphology of COIPV adds challenge to the procedural performance of both CBA and RFA. In the current study, RFAs were performed when the concept of high-power short-duration (15, 16) was validated by a few studies and not recognized widely in clinical practice, thus the operator adopted the conventional low-power approach for ablation. When low-power RF delivery was applied at the posterior aspect and the roof of LA, the operator manipulated the catheter with cautions and dedicated in catheter stability, contact force, and local electrograms at the same time, which significantly lengthened the procedure time. When RF delivery was near the ridge between LIPV and RIPV, good stability of catheter tip was hard to achieve. Thus, separate isolation of the two IPVs was waived and the “tricircle” ablation was performed to surround the common ostium of both IPVs.

For patients who underwent CBA, individualized CB manipulation was needed in the setting of COIPV. In reconstruction images of COIPV, the RIPV was revealed to locate more centrally on the posterior wall and often fused with LIPV in the proximal portion. When the Achieve catheter was hard to reach RIPV, it was sent into LIPV first and followed by the FlexCath sheath. After retreating Achieve slightly while rotating FlexCath posteriorly, then the Achieve could be sent into RIPV.

The large and irregular common ostium of IPVs were hard to engage with 28-mm CB for full-occlusion. Therefore, cryoablation was performed separately in each of IPVs, and the “proximal seal” technique (6) was adopted to prevent CB from being too deep into PVs. Besides, the mismatch between the geometrical shape of CB and the ovoid cross-section of PV antrum could lead to incomplete contact in the cryoablation area. To generate an integrated circumferential ablation area without conduction gaps, the operator performed cryoablation for the upper and lower portions of IPV ostium in separate freezes. The more ovoid IPV ostium became, the more cryo-applications were needed to fill the possible gaps, as shown by the comparison in ostial ovality indexes between subgroups A and B.

Additionally, the frontal orientation of IPVs might affect the manipulation of CB. Greater frontal angles of both IPVs were seen in patients with more cryo-applications in IPVs, although no statistical significance was seen. It was hypothesized that an IPV with a greater degree of frontal orientation was more likely to cause CB to bend and recede, resulting in dealignment between the principal axis of CB and the FlexCath sheath. In this study, the operator sent Achieve catheter deeper into RIPV to support and stabilize the CB, then adjusted the sheath and CB coaxially, and recorded PVPs by slightly retreating Achieve. If it was hard to keep the CB coaxial with sheath, more adjustments and cryo-applications were performed to ensure an efficient contact and to avoid conduction gaps.

### Safety Performance in the Setting of COIPV

It is well-known that the LIPV has a close anatomical relationship with the esophagus. In this study, MDCT scanning showed that eight esophagi stayed in contact with the LIPV, and among them, three esophagi located closely to the junction part of the two IPVs, where might overlap the ablation area. Without using esophageal temperature monitoring, the procedures were performed with great caution and the pantoprazole was routinely applied after the procedure. For RFA, under the premise of PVI, delivering RF energy with moderate time to minimize the damage to the esophagus. For CBA, a distal CB location leads to complications, and it is indicated with a precipitous decline in freezing temperature (< −40°C at 30 s) or a nadir temperature of −55 to −60°C. (6) Thus, the “proximal-seal” technique was routinely adopted and the temperature curve was strictly monitored during cryoablation. Besides, the continuous and repeated cryo-applications at the same site were prohibited to protect the PVs and adjacent structures.

### Clinical Implication

Compared with RFA, the CBA is an efficient, safe, and time-saving technique with similar procedure outcomes. During the freeze cycle, the operator can review the PV venogram to assess the CB occlusion, and meanwhile to plan the next cryoablation site and the target branch of PV for placement of the Achieve catheter (6). Besides, with the help of ICE and new mapping systems, the dependence on fluoroscopy has been greatly reduced, and the CBA technique will become a promising technique in the future.

An assessment of the anatomical characteristics of COIPV is indispensable before the AF procedure. The CBA procedure becomes challenging in the setting of COIPV, especially for those with oval ostium and larger frontal angle of IPVs. A relatively low trans-septal puncture near the limbus of septum is preferable to improve contact between the CB and the inferior aspects of PVs (6), and to allow more space for manipulating CB into IPVs (especially the RIPV). In consideration of the size and irregularity of common ostium, a separate freeze for each of the IPVs becomes a better choice to avoid conduction gaps. Although the IPV ostium with higher ovality is hard to engage with CB, the cryoablation can be performed with segmental freezing strategy, which applies to freezing separately for the upper and lower portions of IPV ostium. It is unnecessary to pursue a “single-shot freezing” with full occlusion for IPV ostium, and there is no need for special skills such as “pull-down,” “hockey stick,” and “big-loop” technique (17).

### Limitations

First, this is a single-center study with a small sample size, which limits the reliability of conclusions in comparison between RFA and CBA. Furthermore, due to the rarity of COIPV, the IPV with small common ostium, which might be more suitable for “single-shot” cryo-application with full occlusion by 28-mm CB, was not encountered, and all CBA cases in this study underwent separate cryoablation for each of the IPVs. An appropriate choice of cryoablation strategy for COIPV with different ostium sizes still remains unclear. Additionally, the ablation technique and strategy were at the discretion of the operator. For RFA, the strategy of high-power short-duration was not used although it had similar efficacy, safety, and shorter procedure time in AF ablation (18–20). For CBA, the total number of cryo-applications and procedure time might be affected by operator's proficiency and CB performance. Thus, further randomized trials with enough cases and novel ablation strategies are needed to compare the CBA and RFA. Second, no esophageal temperature monitoring nor post-procedure endoscope was performed to evaluate the safety of CBA in COIPV. Third, except for two redo-procedures, the electrophysiologic screening was not routinely performed during follow-up, which may underestimate the concealed reconnection of PVs. Besides, the two redo-procedures were performed with point-by-point mapping only. Without using voltage mapping and ablation index, there is a shortage in quantitative evaluation of lesion extent and the efficacy of cryoablation. Finally, the possibility of asymptomatic and undetected AAs still exists in spite of regular rhythm screening in the follow-up period. The use of implantable monitoring devices can be reliable to compensate for this flaw.

## Data Availability Statement

The original contributions presented in the study are included in the article/Supplementary Material, further inquiries can be directed to the corresponding author.

## Ethics Statement

The studies involving human participants were reviewed and approved by Ethics Committee of Fuwai Hospital. The patients/participants provided their written informed consent to participate in this study.

## Author Contributions

X-gG, QS, and JM contributed to the conception of the study. J-hL performed the data analyses and wrote the manuscript. H-yX, Y-qC, and Z-jC helped perform the analysis with constructive discussions. All authors contributed to the article and approved the submitted version.

## Funding

This study was supported by the National Natural Science Foundation of China (Grant No. 81670309).

## Conflict of Interest

The authors declare that the research was conducted in the absence of any commercial or financial relationships that could be construed as a potential conflict of interest.

## Publisher's Note

All claims expressed in this article are solely those of the authors and do not necessarily represent those of their affiliated organizations, or those of the publisher, the editors and the reviewers. Any product that may be evaluated in this article, or claim that may be made by its manufacturer, is not guaranteed or endorsed by the publisher.
