# Supplementary material for: Comparison of Using Second-Generation Cryoballoon and Radiofrequency Catheter for Atrial Fibrillation Ablation in Patients With the Common Ostium of Inferior Pulmonary Veins
Source: Front Cardiovasc Med. 2022 Jan 11;8:794834. doi: 10.3389/fcvm.2021.794834 (PMC8787139; doi:10.3389/fcvm.2021.794834)
Supplement: Supplementary file 1 [file Data_Sheet_1.PDF]

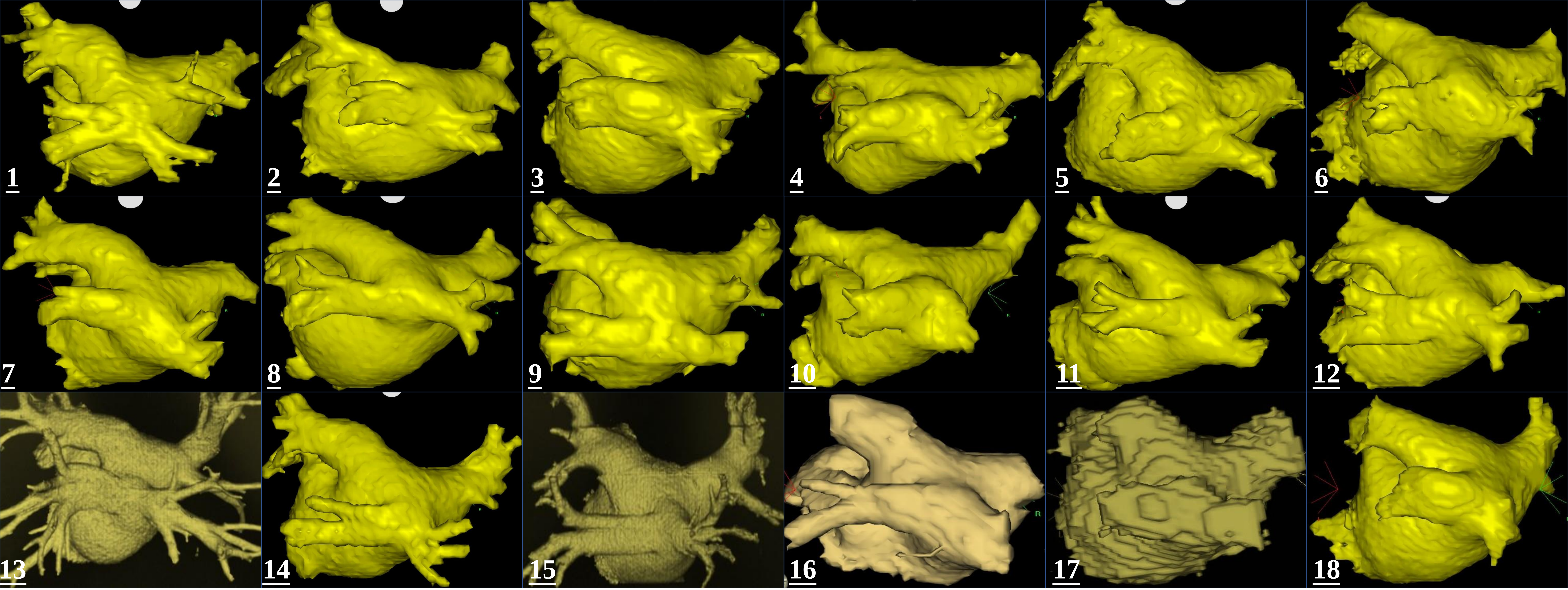

Supplemental Figure S1

Three-dimensional computer tomography reconstruction images on posterior-anterior view of left atrium and pulmonary veins of the enrolled 18 patients. Patient #1 to #10 were in CBA group and patient #11 to #18 were in RFA group.

CBA, cryoballoon ablation; RFA, radiofrequency ablation.
