# Supplementary material for: Comparison of Using Second-Generation Cryoballoon and Radiofrequency Catheter for Atrial Fibrillation Ablation in Patients With the Common Ostium of Inferior Pulmonary Veins
Source: Front Cardiovasc Med. 2022 Jan 11;8:794834. doi: 10.3389/fcvm.2021.794834 (PMC8787139; doi:10.3389/fcvm.2021.794834)
Supplement: Supplementary file 2 [file Data_Sheet_2.PDF]

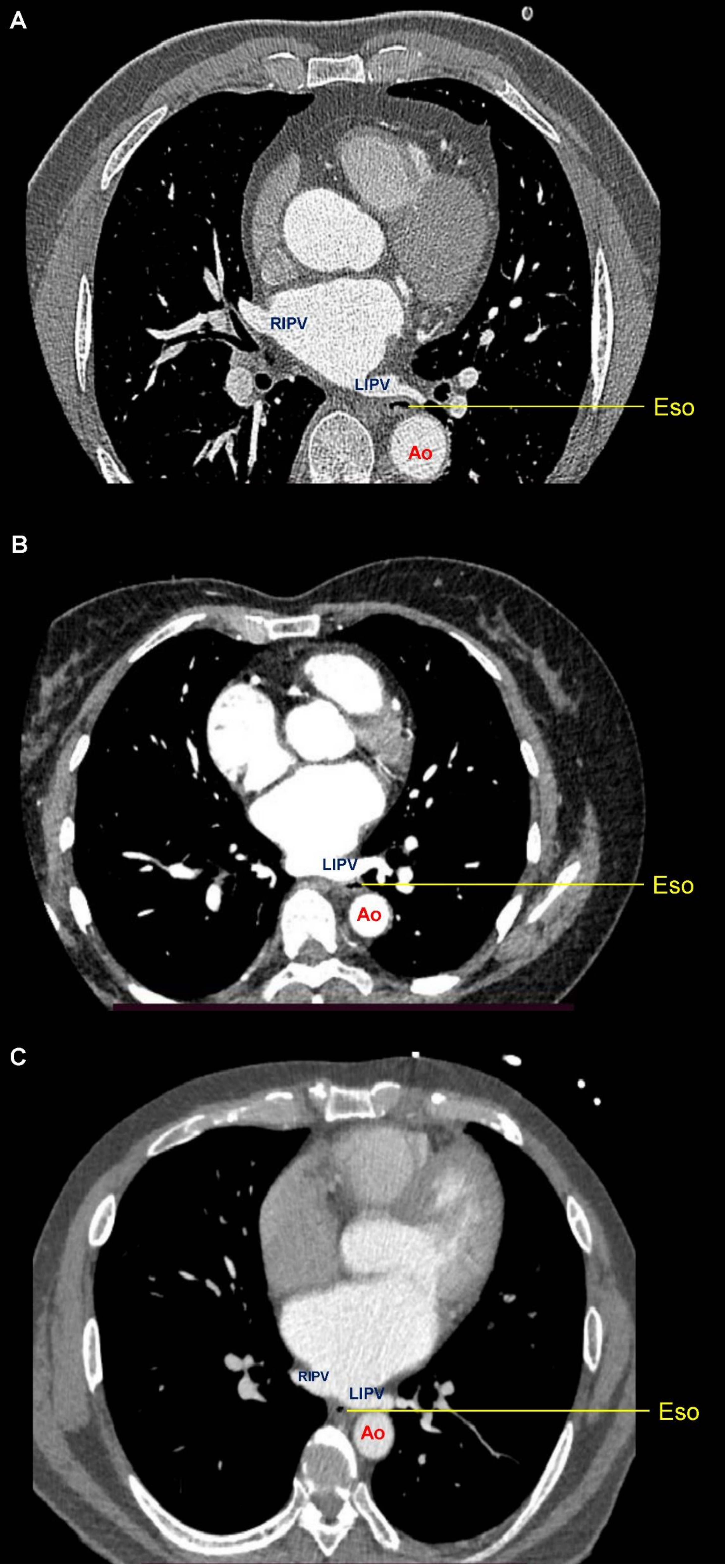

Supplemental Figure 2

MDCT scanning showed that the esophagus (A) stayed away from LIPV, and (B) located near the main branch of LIPV but away from the ostium, and (C) located closely to the posterior junction part of LIPV and RIPV.

Ao, aorta; Eso, esophagus; LIPV, left inferior pulmonary vein; MDCT, multidetector computed tomography; RIPV, right inferior pulmonary vein.
